# Cellular localization of nucleolin determines the prognosis in cancers: a meta-analysis

**DOI:** 10.1007/s00109-022-02228-w

**Published:** 2022-07-21

**Authors:** Supaporn Yangngam, Jaturawitt Prasopsiri, Phimmada Hatthakarnkul, Suyanee Thongchot, Peti Thuwajit, Pa-thai Yenchitsomanus, Joanne Edwards, Chanitra Thuwajit

**Affiliations:** 1 Department of Immunology, Faculty of Medicine Siriraj Hospital, Mahidol University, Bangkok, 10700 Thailand; 2 Biomedical Sciences Graduate Program, Faculty of Medicine Siriraj Hospital, Mahidol University, Bangkok, 10700 Thailand; 3 Institute of Cancer Sciences, University of Glasgow, Wolfson Wohl Cancer Research Centre, Garscube Estate, Glasgow, G61 1QH UK; 4 Siriraj Center of Research Excellence for Cancer Immunotherapy (SiCORE-CIT), Research Department, Faculty of Medicine Siriraj Hospital, Mahidol University, Bangkok, 10700 Thailand

**Keywords:** Nucleolin, Cancer, Meta-analysis, Prognostic marker, Localization

## Abstract

Nucleolin (NCL) is a multifunctional protein expressed in the nucleus, cytoplasm, and cell membrane. Overexpression of NCL has a controversial role as a poor prognostic marker in cancers. In this study, a meta-analysis was performed to evaluate the prognostic value of NCL in different subcellular localizations (cytoplasmic (CyNCL) and nuclear (NuNCL)) across a range of cancers. PubMed was searched for relevant publications. Data were extracted and analyzed from 12 studies involving 1221 patients with eight cancer types. The results revealed high total NCL was significantly associated with poor overall survival (OS) (HR = 2.85 (1.94, 4.91), *p* < 0.00001, *I*^2^ = 59%) and short disease-free survival (DFS) (HR = 3.57 (2.76, 4.62), *p* < 0.00001, *I*^2^ = 2%). High CyNCL was significantly associated with poor OS (HR = 4.32 (3.01, 6.19), *p* < 0.00001, *I*^2^ = 0%) and short DFS (HR = 3.00 (2.17, 4.15), *p* < 0.00001, *I*^2^ = 0%). In contrast, high NuNCL correlated with increased patient OS (HR = 0.42 (0.20, 0.86), *p* = 0.02, *I*^2^ = 66%), with no significant correlation to DFS observed (HR = 0.46 (0.19, 1.14), *p* = 0.09, *I*^2^ = 57%). This study supports the role of subcellular NCL as a poor prognostic cancer biomarker.

## Introduction

Nucleolin (NCL) is a eukaryotic nucleolar phosphoprotein involved in the synthesis and maturation of ribosomes, gene silencing, senescence, cytokinesis, cell proliferation, and growth [1–5]. NCL is predominately located in dense fibrillar regions of the nucleolus, being observed less frequently in the cytoplasm and membrane [6–10]. NCL was reported to be expressed in the nuclear fraction of normal human mammary epithelial cells, with only low levels observed in the cytoplasm [11]. Moreover, nuclear NCL (NuNCL) was observed in a variety of normal human tissues, including hepatocytes and cholangiocytes in the liver, endocrine cells and exocrine glandular cells in the pancreas, respiratory epithelial cells in the nasopharynx and bronchus, squamous epithelial cells in the esophagus, and glandular cells in the stomach, according to immunohistochemistry (IHC) (data obtained from The Human Protein Atlas (https://www.proteinatlas.org/)) [12].

In cancer, NCL is reported to be overexpressed with altered subcellular localization. Immune fluorescent and cell fractionation studies demonstrated that an increase in cytoplasmic NCL and a decrease in nuclear NCL were observed in the MCF-7 breast cancer cell line compared to a normal breast cancer cell line [11]. In human cancer specimens, IHC studies report that an increase in expression of NCL (without defining the subcellular localization) was associated with poor prognosis in pediatric and adult ependymoma [13, 14], hepatocellular carcinoma [15], non-small cell lung cancer [16], esophageal squamous cell carcinomas [17], and B cell lymphoma [18]. In contrast, no significant association with NCL expression and prognosis was reported in studies of ependymoma [14] hepatocellular carcinoma [19] and pancreatic ductal adenocarcinoma [20]. High NCL expression in the cytoplasm was associated with a poor prognosis in gastric cancer patients [21] and endometrial cancer [22] whereas the high nuclear NCL expression was an independent good prognostic marker in gastric cancer, endometrial carcinoma, and pancreatic ductal adenocarcinoma [21–23]. However, when subcellular localization is considered, NuNCL was reported to be significantly higher in fibrosarcoma, chondrosarcoma, liposarcoma, rhabdomyosarcoma, testicular tumor, and cutaneous melanocytic lesion tissues than in normal adjacent tissues, and the mRNA expression level has been linked to poor survival in patients [24–26]. On balance, there is a body of evidence in the literature that supports NCL as a potential prognostic marker in cancer; however, whether its subcellular expression levels are associated with good or poor prognosis requires further clarification.

**Fig. 1:**
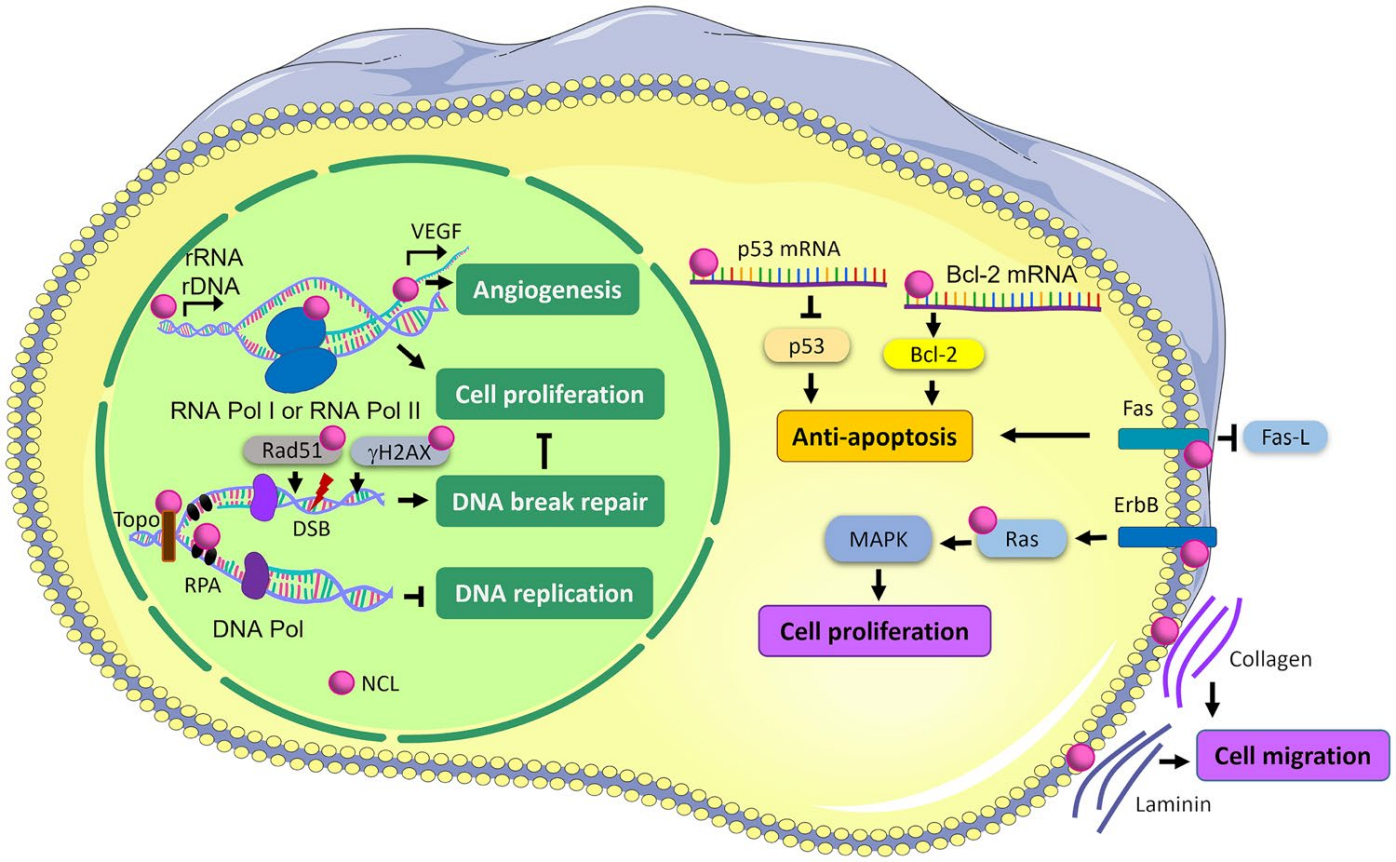
Subcellular NCL functions in cancer cell. NCL: nucleolin, rRNA: ribosomal ribonucleic acid, rDNA: ribosomal deoxyribonucleic acid, RNA Pol I and II: RNA polymerase I and II, VEGF: vascular endothelial growth factor, Topo: topoisomerase, RPA: replication protein A, DNA Pol: DNA polymerase, DSB: DNA double-strand break, Rad51: DNA repair protein RAD51 homolog 1, γH2AX: Ser-139 phosphorylation of H2A histone family member X, Bcl-2: B-cell lymphoma 2; Fas: Fas cell surface death receptor, Fas-L: Fas cell death ligand, Ras: Rat sarcoma, ErbB: receptor tyrosine-protein kinase erbB, MAPK: mitogen-activated protein kinase

As NCL has distinct functions depending on different cellular compartments in cancer cells, it is not surprising that intracellular localization is associated with varying patient outcomes [27]. NuNCL can regulate ribosomal DNA (rDNA) and rRNA syntheses, ribosome assembly, and the transcriptional activities of RNA polymerases (RNA pol) I and II [27–29] leading to cell proliferation (Fig. 1). It can bind with vascular endothelial growth factor (VEGF) promotor and increases the expression of VEGF which eventually promotes angiogenesis [30]. Furthermore, NuNCL has been implicated in the regulation of miRNAs, and transcription factors such as TBX3 that are involved in tumorigenesis promote cell proliferation and cell migration [24, 31, 32]. However, NuNCL has also been reported to bind with replication protein A (RPA) and prevent DNA replication by inhibiting RPA DNA replication initiation and elongation in human bone osteosarcoma epithelial cells [33]. Moreover, NCL binds to several DNA repair proteins such as topoisomerase (Topo) in U-937 leukemic cell [34] and Rad51 in human fibrosarcoma cells [35]; Ser-139 phosphorylation of H2A histone family member X (γH2AX) in HeLa cells [36] facilitates double-stranded break DNA repair machinery which then can delay cell proliferation. In addition, NCL promoted cisplatin resistance via the YB1-MDR pathway in cervical cancer which has been reported through the NCL-mediated cell proliferation leading to the attenuation of cancer cell sensitivity to cisplatin. This overexpression of NCL was associated with increased multidrug resistance (*MDR1*) gene expression resulting to the increment of drug efflux via transcription factor YB1 [37].

In contrast, cytoplasmic NCL (CyNCL) and membranous NCL (MemNCL) are linked to proliferation, anti-apoptosis, and migration [9, 27]. CyNCL inhibits apoptosis by interacting with the 5′ UTR of p53 resulting in decreasing p53 translation [38] and increasing the stability of anti-apoptotic *Bcl-2* mRNA in leukemic cells [39] (Fig. 1). MemNCL interacts with Fas receptor to prevent Fas-induced apoptosis activated by Fas-ligand (FAS-L) in B cell lymphoma cells [40]. CyNCL and MemNCL have been associated with an anti-apoptotic phenotype in cancer cells. Moreover, MemNCL has been reported to promote cell proliferation and tumor growth by binding to Ras and activating the Ras/MAPK cascade as the result of erythroblastic leukemia viral oncogene homolog (ErbB) receptor activation in colon cancer cells and prostate cancer cells [41]. Additionally, the MemNCL induced by VEGF via PI3K/Akt pathway in colorectal cancer [42] can act as an adhesion molecule to interact with collagen and laminin leading to cancer cell migration [43] (Fig. 1).

Based on the previously published evidence, NCL may be a potential cancer marker, and its subcellular localization may be useful to determine the prognosis of the cancer patients. Herein, the purpose of this study is to evaluate the prognostic value of NCL in varying subcellular locations in cancers using meta-analysis to propose the specific subcellular NCL as a potent prognostic marker in the patients.

## Materials and methods

### Literature search

An online literature search (PubMed, https://pubmed.ncbi.nlm.nih.gov/) was conducted between the 24th of October 2021 and the 27th of November 2021 to assess the level of NCL and its clinicopathological correlation in several cancers. The PubMed literature was filtered using the keywords “nucleolin” or “NCL” in combination with “expression” and “cancer”.

### Inclusion and exclusion criteria

The studies were considered eligible if they met the following criteria: (1) NCL was detected in cancer tissues using immunohistochemistry (IHC) and (2) the hazard ratio (HR) for survival rate of either overall survival (OS) or disease-free survival (DFS) was calculated, and the 95% confidence intervals (*CI*) were provided. The following studies were excluded: (1) non-human studies; (2) non-English language studies; (3) in vitro studies; and (4) articles from which the relevant data could not be extracted.

### Data extraction

Each eligible study was reviewed by two investigators (JP and SY), and the following information was extracted: the first author’s name, publication date, country, number of participants, age, gender, the percentage of NCL positivity, and the cellular localization of NCL. Furthermore, if the HR and 95% *CI* were reported in the text or survival table, they were collected. When it was not possible to extract HR directly from the article, Kaplan–Meier (KM) curves were used to estimate HR following the method of Tierney et al. [44]. Briefly, the percentage of survival over the interval times was extracted from the KM curve using the GetData Graph Digitizer 2.26 (http://getdata-graph-digitizer.com/download.php) and input into the established spreadsheet to generate the estimate HR and 95% *CI*. Disagreements between reviewers were settled through discussion.

### Statistical analysis

The association of NCL expression in cancer patient survival time was evaluated by HR with an estimate of 95% *CI*. If the articles presented both univariate (UV) and multivariate analysis (MV), MV analysis was preferred. The heterogeneity of the data from eligible studies was evaluated by *I*^2^ statistic, which is a quantitative measure of inconsistency across studies using a random effect model. The *I*^2^ varies from 0 (no observed heterogeneity) to 100% (maximal heterogeneity). *I*^2^ value of more than 50% was considered to represent substantial heterogeneity among studies. Statistical significance was defined as *p*-value < 0.05. All analyses were performed using Review Manager (RevMan) version 5.4 (The Nordic Cochrane Centre, The Cochrane Collaboration, Copenhagen, Denmark).

### IHC staining of NCL and scoring in breast cancer tissues

NCL was detected on the paraffin-embedded tumor microarray of 147 TNBC cases under the approval of sample collection by Siriraj Institutional Review Board (COA no. Si 580/2018). The staining protocol was as reported previously [45]. In brief, 4-µm-thick sections were incubated with anti-NCL antibody (#14574, Cell Signaling Technology, Inc) in a humidified chamber at 4 °C and then with rabbit Envision System HRP-labeled polymer IHC secondary antibody (K4003, DAKO) for 30 min at RT (room temperature). The peroxidase activity was visualized with diaminobenzidine (DAB) solution and counterstained by hematoxylin. The staining proteins were quantitatively scored by scanning the slides with 3DHistech Ltd. CaseViewer/QuantCenter software 2.4.0. (Sysmex) and scored based on: 0x% not stained + 1x% weakly stained + 2x% moderately stained + 3x% strongly stained. This gave a range of scores from 0 to 300 with nuclear, cytoplasmic, and membrane tumor-specific staining scored separately. The expression of protein at each cellular compartment was divided into low and high using the cutoff point calculated by RStudio version 2022.2.0.443 (Integrated Development for R. RStudio, PBC, Boston, MA URL http://www.rstudio.com/)*.*

## Results

### Study selection and characteristic

**Fig. 2:**
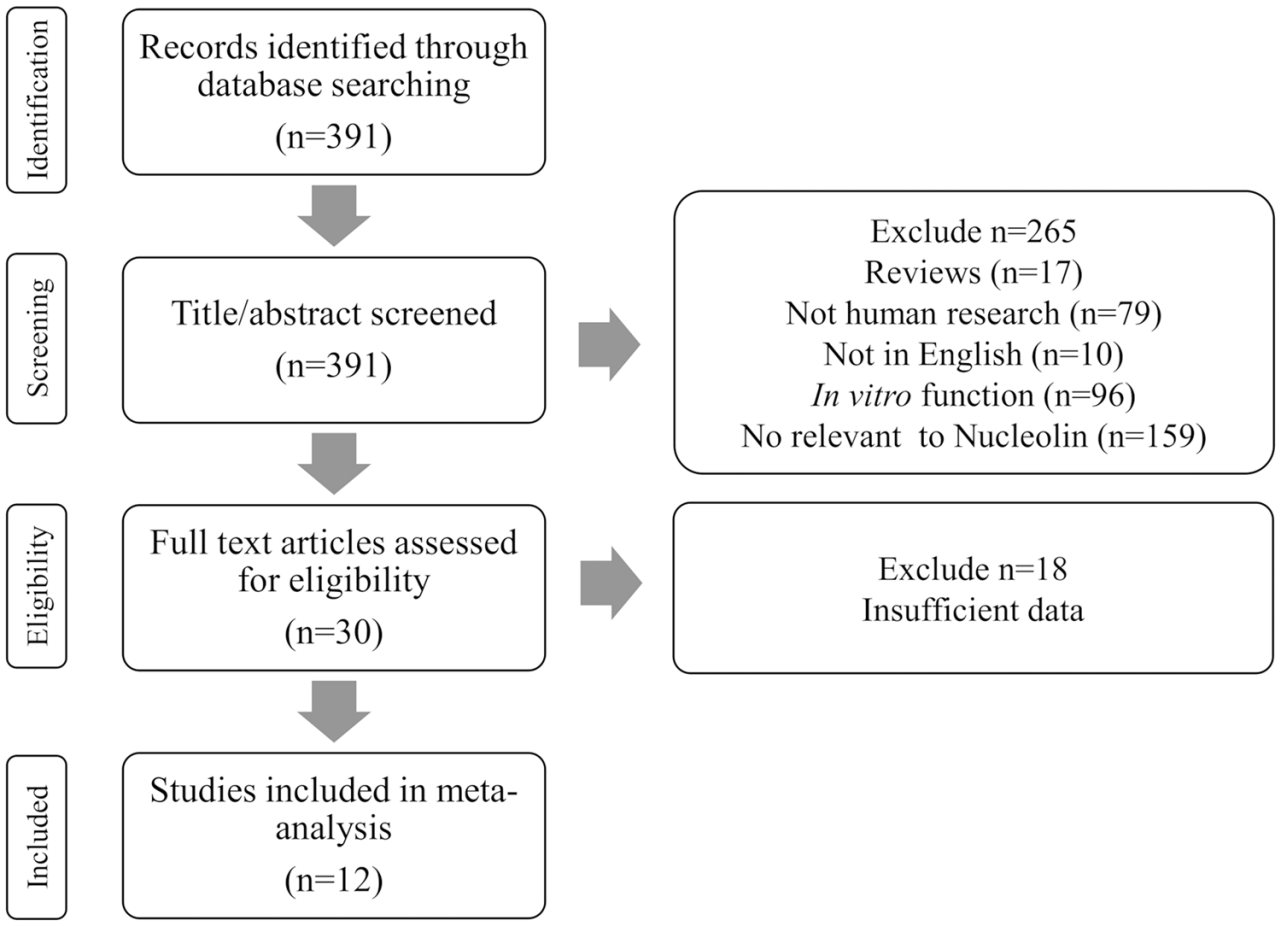
Flowchart of the literature search and study selection procedure

Following the initial PubMed search, 391 papers were identified. Based on their titles and content of abstracts, 265 articles were removed including 17 review articles, 79 non-human research, 10 non-English articles, 96 in vitro functional tests, and 159 irrelevant to NCL. The remaining 30 papers were identified through full text. Eighteen papers were excluded due to insufficient data for example no data of OS or DFS and did not use IHC for NCL expression measurement. Finally, 12 studies met the inclusion criteria for the meta-analysis (Fig. 2 and Table 1).

**Table 1:** Characteristic of the eligible studies for meta-analysis in this study

**Ref**	**Cancer (*n*)**	**NCL**						**MV**	**S**	**HR (95%*CI*)**	***p*-value**
		**Total (*n*)**		**Cy (*n*)**		**Nu (*n*)**					
		**H**	**L**	**H**	**L**	**H**	**L**				
[13]	Pediatric intracranial EP (64)	52 (81%)	12 (19%)	N/A	N/A	N/A	N/A	Surgical resection and total NCL	DFS	MV 6.25 (1.61, 24.21)	0.008
[14]	Childhood EP (60)	40 (67%)	20 (33%)	N/A	N/A	N/A	N/A	Not significant	OS	UV 2.1 (0.8, 5.6)	0.130
										MV 1.3 (0.6, 2.6)	0.539
									DFS	UV 3.0 (1.4, 6.6)	0.006
										MV 1.9 (0.9, 3.8)	0.090
[46]	EP (174)	116 (67%)	58 (33%)	N/A	N/A	N/A	N/A	Age and total NCL	DFS	MV 8.73 (1.69, 45.06)	0.010
[15]	HCC (130)	78 (60%)	52 (40%)	N/A	N/A	N/A	N/A	Serum AFP, tumor stage, total NCL	OS	MV 3.87 (1.68, 8.39)	0.010
									DFS	MV 3.696 (1.662, 8.138)	0.010
[19]	HCC (50)	32 (64%)	18 (36%)	N/A	N/A	N/A	N/A	N/A	OS*	UV 1.95 (0.99, 3.84)	0.053
[16]	NSCLC (92)	57 (62%)	35 (38%)	N/A	N/A	N/A	N/A	N/A	OS*	UV 2.46 (1.14, 5.31)	0.0218
[20]	PDAC (47)	35 (75%)	12 (25%)	N/A	N/A	N/A	N/A	N/A	OS*	UV 2.05 (1.00, 4.20)	0.050
[18]	BCL (104)	58 (56%)	46 (44%)	N/A	N/A	N/A	N/A	N/A	OS*	UV 4.84 (2.62, 8.94)	< 0.0001
[47]	NSCLC (225)	117 (52%)	108 (48%)	116 (52%)	109 (48%)	71 (32%)	154 (68%)	Histological type, pathological stage, total NCL, CyNCL, and NuNCL	OS*	Total; UV 4.59 (3.15, 6.69)	< 0.0001
										Cy; UV 4.39 (3.02, 6.39)	< 0.0001
										Nu; UV 0.67 (0.46, 0.98)	0.0369
									DFS*	Total; UV 3.45 (2.43, 4.90)	< 0.0001
										Cy; UV 2.97 (2.12, 4.16)	< 0.0001
										Nu; UV 0.63 (0.45, 0.88)	0.0071
[21]	GC (124)	N/A	N/A	22 (18%)	102 (82%)	85 (69%)	39 (31%)	NuNCL and CyNCL expressions	OS	Cy; UV 5.557 (2.598, 11.89)	< 0.0001
										Cy; MV 3.578 (0.5–16.42)	0.0485
										Nu; UV 0.351 (0.165–0.747)	0.0075
										Nu; MV 0.113 (0.023–0.552)	0.0063
[22]	EC (82)	N/A	N/A	34 (42%)	48 (59%)	55 (67%)	27 (33%)	Stage, NuNCL, and CyNCL	DFS	Cy; UV 3.398 (1.046, 11.038)	0.042
										Cy; MV 3.377 (1.029, 11.187)	0.046
										Nu; UV 0.178 (0.055, 0.580)	0.004
										Nu; MV 0.233 (0.068, 0.796)	0.020
[23]	PDAC (69)	N/A	N/A	N/A	N/A	35 (51%)	34 (49%)	Tumor differentiation and Nu NCL	OS	UV 0.48 (0.25, 0.93)	0.030
										MV 0.39 (0.20, 0.79)	0.010

*Cy* cytoplasm, *H* high, *L* low, *Nu* nucleus, *NCL* nucleolin, *CyNCL* cytoplasmic NCL, *NuNCL* nuclear NCL, *UV* univariate analysis, *MV* multivariate analysis, *S* survival, *EP* ependymoma, *HCC* hepatocellular carcinoma, *EC* endometrial cancer, *NSCLC* non-small cell lung cancer, *GC* gastric cancer, *PDAC* pancreatic adenocarcinoma, *BCL* B cell lymphoma, *N/A* non-applicable, *OS* overall survival, *DFS* disease-free survival, *estimated HR from estimation method

### Study characteristics

The characters of the 12 papers included in the study were listed (Table 1). The included studies were published between 2008 and 2021. The OS was reported in 9 studies, and DFS was reported in 6 articles. The 12 papers include 3 studies on pediatric and adult ependymoma [13, 14, 46], two on hepatocellular carcinoma [15, 19], two on pancreatic ductal carcinoma [20, 23], two on non-small cell lung cancer [16, 47], one on endometrial cancer [22], one on gastric cancer [21], and one on B-cell lymphoma [18].

The antibodies used in IHC for NCL detection in the cancer tissues were from different clones with a variety of binding epitopes binding sites, binding to amino acids 2–17 [16] and 271–520 [18] of the 710 amino acids NCL protein or undefined NCL epitope using whole human NCL protein from Raji cell extract as the immunogen for the antibody production [13–15, 21–23, 46] or antibody clones that did not provide the antigen binding site [19, 20, 47]. The cellular localization of NCL expression was divided into three groups including expression in all cellular compartments (total NCL), CyNCL, and NuNCL (Table 1). According to these three subcellular classifications, only 8 papers reported total NCL with a total of 721 patients [13–16, 18–20, 46]; 1 paper reported total NCL, CyNCL, and NuNCL (225 patients) [47]; 2 papers reported CyNCL and NuNCL (206 patients) [21, 22]; and 1 paper reported only NuNCL (69 patients) [23]. Notably, no papers reported only CyNCL. For total NCL (from the articles which the authors did not provide the localization of NCL results in their work), we had 9 papers to be analyzed which the figure results confirmed the combination of both CyNCL and NuNCL [13–16, 18–20, 46, 47]. For CyNCL, 3 papers were provided [21, 22, 47], whereas 4 papers offered data of NuNCL [21–23, 47]. Three papers directly provided survival data of only DFS [13, 22, 46]. Six papers showed only patient OS data, of which two of them directly presented HR and 95% *CI* [21, 23], while the remaining 4 papers, the estimated HR and 95% *CI* were performed [16, 18–20]. Three papers reported both OS and DFS [14, 15, 47], of which 1 estimated HR and 95% *CI* [47].

### The impact of total NCL, CyNCL, and NuNCL on cancer patient OS

**Fig. 3:**
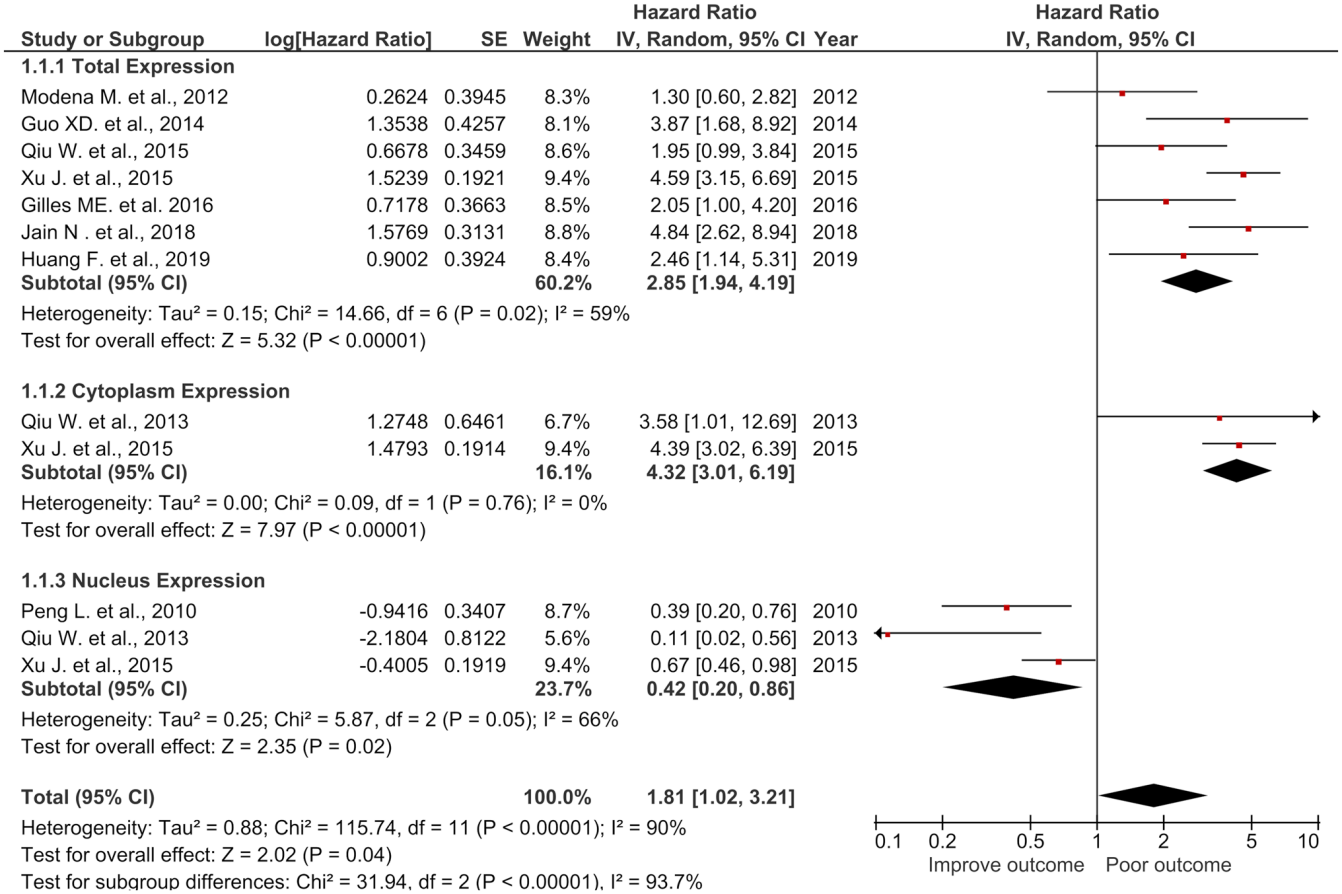
Forest plot of HR and 95% *CI* for the association of the total, cytoplasmic, nuclear, and the combined (total + cytoplasmic + nuclear) NCL with OS of the cancer patients

The meta-analysis was performed on 7 studies for total NCL expression (708 patients), 2 studies for cytoplasmic NCL expression (349 patients), and 3 articles contained 418 patients for NuNCL expression assessing the association of each NCL localization with patient OS. The results from total NCL exression group showed that high NCL expression had a significant association with poor OS (HR = 2.85, 95% *CI* = (1.94, 4.19), *p* < 0.00001) with heterogeneity (*I*^2^ = 59%) (Fig. 3). High expression of CyNCL was significantly associated with poor OS in the patients (HR = 4.32, 95% *CI* = (3.01, 6.19), *p* < 0.00001) without heterogeneity (*I*^2^ = 0%). In contrast with total NCL and CyNCL, high expression of NuNCL was significantly associated with improved patient outcome (HR = 0.42, 95% *CI* = (0.2, 0.86), *p* = 0.02) with heteroginiety (*I*^2^ = 66%) (Fig. 3). The combined results of the combined total NCL, CyNCL, and NuNCL still revealed as the poor prognostic marker with HR = 1.81 (95% *CI* = 1.02, 3.21, *p* = 0.04, *I*^2^ = 90%) which notably was lower than that of total NCL alone (HR = 2.85) or of CyNCL alone (HR = 4.32). The results indicated that CyNCL had the significant impact on patient survival as a poor prognostic marker followed by the total NCL. Of note, the combined NCL (total, CyNCL, and NuNCL) showed no applicable used as it reduced the prediction of short patient OS. In contrast, NuNCL revealed the impact of a predictive marker for long patient OS with statistical sifgnificance.

### High total NCL and high CyNCL were associated with poor OS in cancer patients

**Fig. 4:**
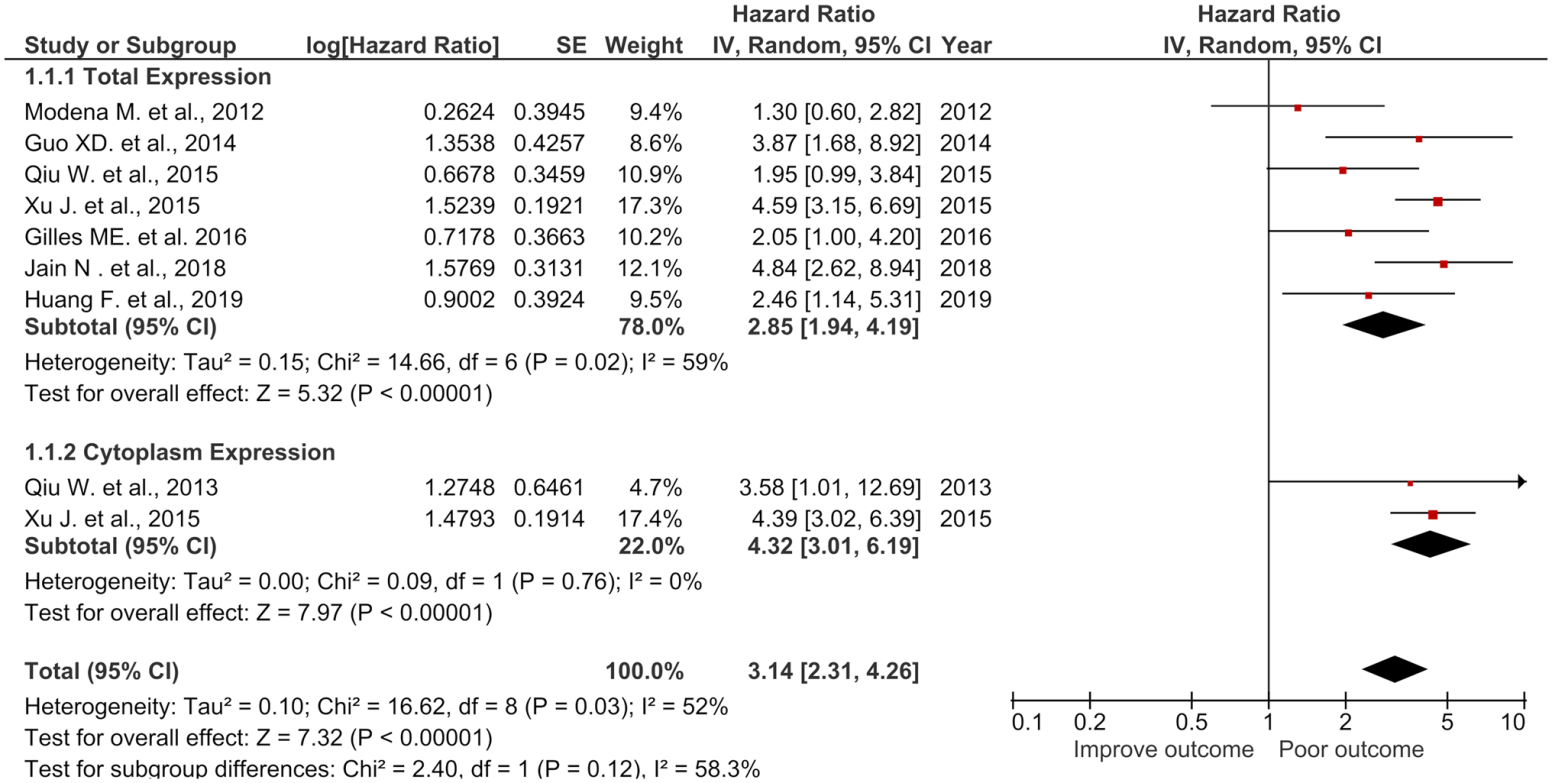
Forest plot of HR and 95% *CI* for the association of the total, cytoplasmic, and the combined (total + cytoplasmic) NCL with OS of the cancer patients

As both total NCL and CyNCL were associated with poor OS in the patients, their combined HR was determined. The results showed that the combined HR of the total NCL and CyNCL was associated with poor OS (HR = 3.14, 95% *CI* = (2.31, 4.26), *p* < 0.00001, *I*^2^ = 0%). This HR was lower than that of CyNCL (HR = 4.32), but higher than that of total NCL (HR = 2.85) (Fig. 4). These results may suggest the NCL in the cytoplasm as the best prognostic NCL marker for shorter OS.

### Impact of total, CyNCL, and NuNCL expressions on DFS of cancer patients

**Fig. 5:**
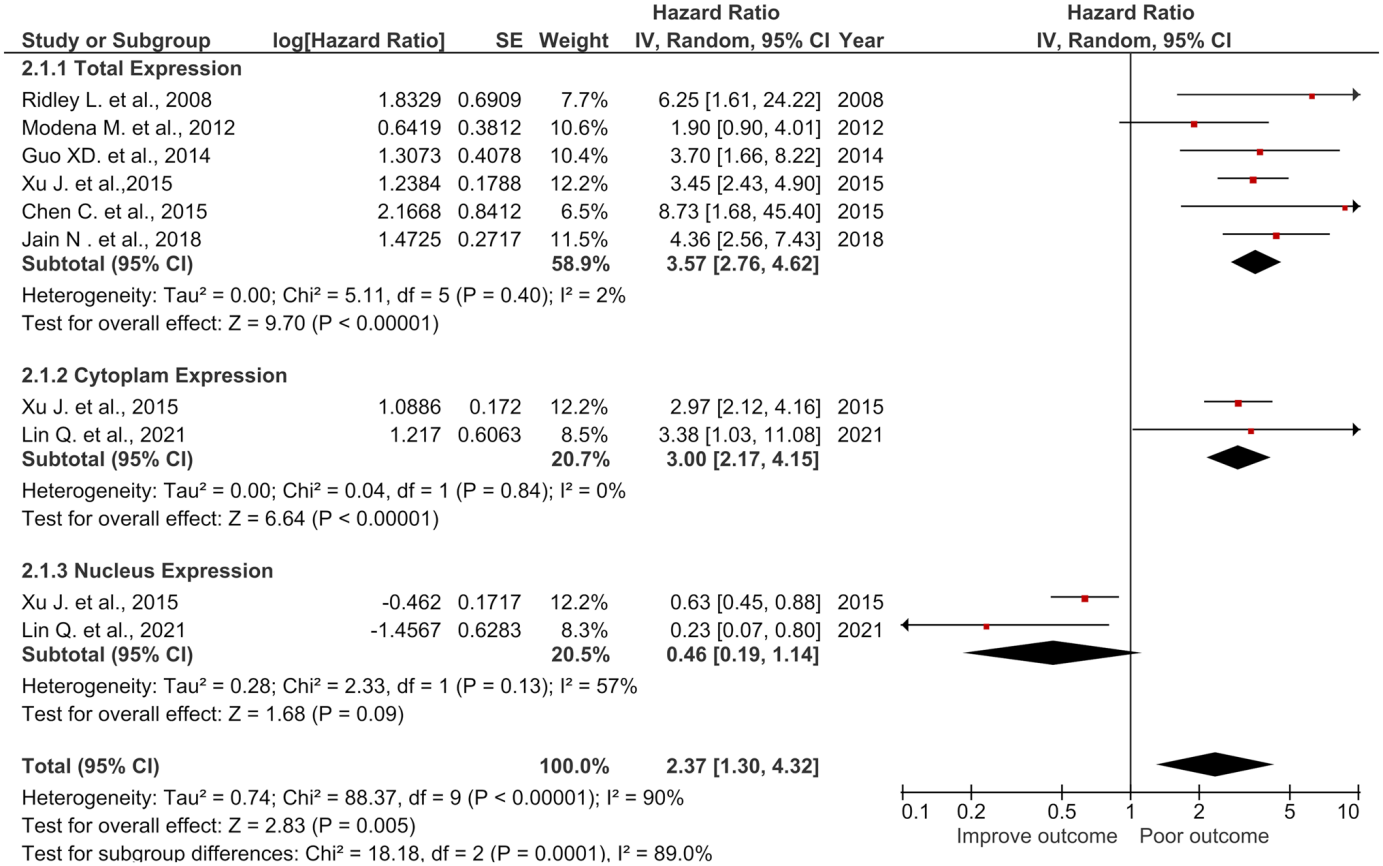
Forest plot of HR and 95% *CI* for the association of the total, cytoplasmic, nuclear, and the combined (total + cytoplasmic + nuclear) NCL with DFS of the cancer patients

Six articles, consisting of 747 patients for total NCL expression; 2 articles (307 samples) from cytoplasmic expression; and 2 articles (357 patients) from nuclear expression were included to determine the effect of total NCL, CyNCL, and NuNCL on DFS (Fig. 5). High expression of total NCL showed significant association with poor DFS (HR = 3.57, 95% *CI* = (2.76, 4.62), *p* < 0.00001) with homogeniety (*I*^2^ = 2%). In the same trend, high CyNCL was significantly associated with poor DFS (HR = 3.00, 95% *CI* = (2.17, 4.15), *p* < 0.00001) with non-heterogeneity (*I*^2^ = 0%) whereas high expression of NCL in the nucleus has no significant corelattion with DFS in the patients (HR = 0.46, 95% *CI* = (0.19, 1.14), *p* = 0.09) with heterogeniety (*I*^2^ = 57%) (Fig. 5). In the similar manner to OS, the combined HR (total NCL, CyNCL, and NuNCL) had the reduced HR compared to using either total NCL or CyNCL for DFS (HR = 2.37, 95% *CI* = (1.30, 4.32), *p* = 0.005) with heterogeneity (*I*^2^ = 90%) (Fig. 5). The results indicated that total NCL and CyNCL may predict short DFS whereas NuNCL would be a predictive marker for long DFS in patients; however, this was not statistically significant.

### High total NCL and high CyNCL were associated with poor DFS in cancer patients

**Fig. 6:**
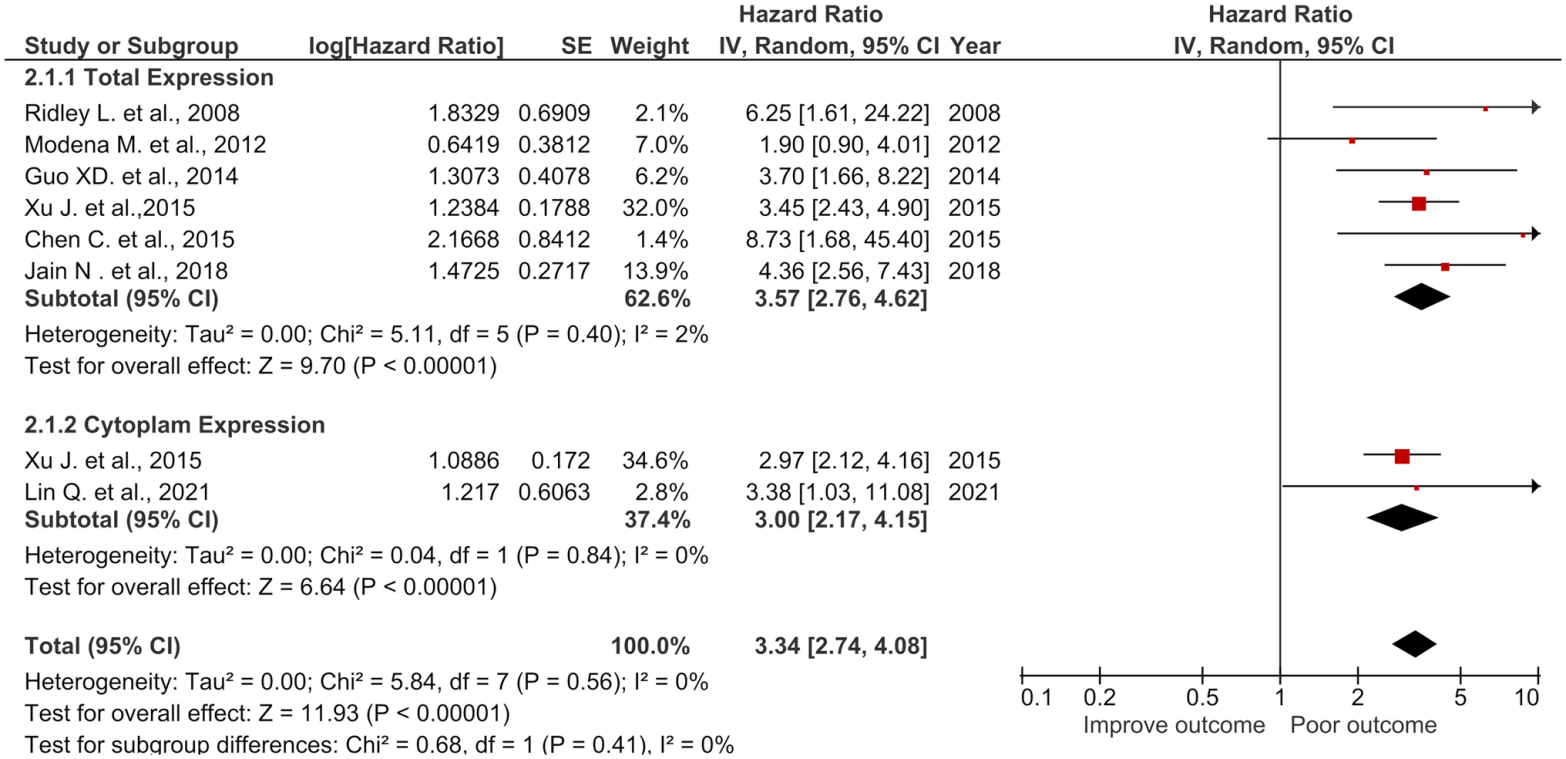
Forest plot of HR and 95% *CI* for the association of total, cytoplasmic, and the combined (total + cytoplasmic) NCL with DFS of the cancer patients

The combined HR and 95% *CI* of total NCL and CyNCL significantly correlated with poor DFS with HR of 3.34 (95% *CI* = 2.74, 4.08) with statistical significance (*p* < 0.00001) without heterogeneity (*I*^2^ = 0%) (Fig. 6). The total NCL (HR = 3.57) and CyNCL (HR = 3.00) showed almost the same HR to that of the combined HR as being the poor prognostic markers for patient DFS time.

### High CyNCL was associated with poor OS in triple-negative breast cancer (TNBC) patients

**Fig. 7:**
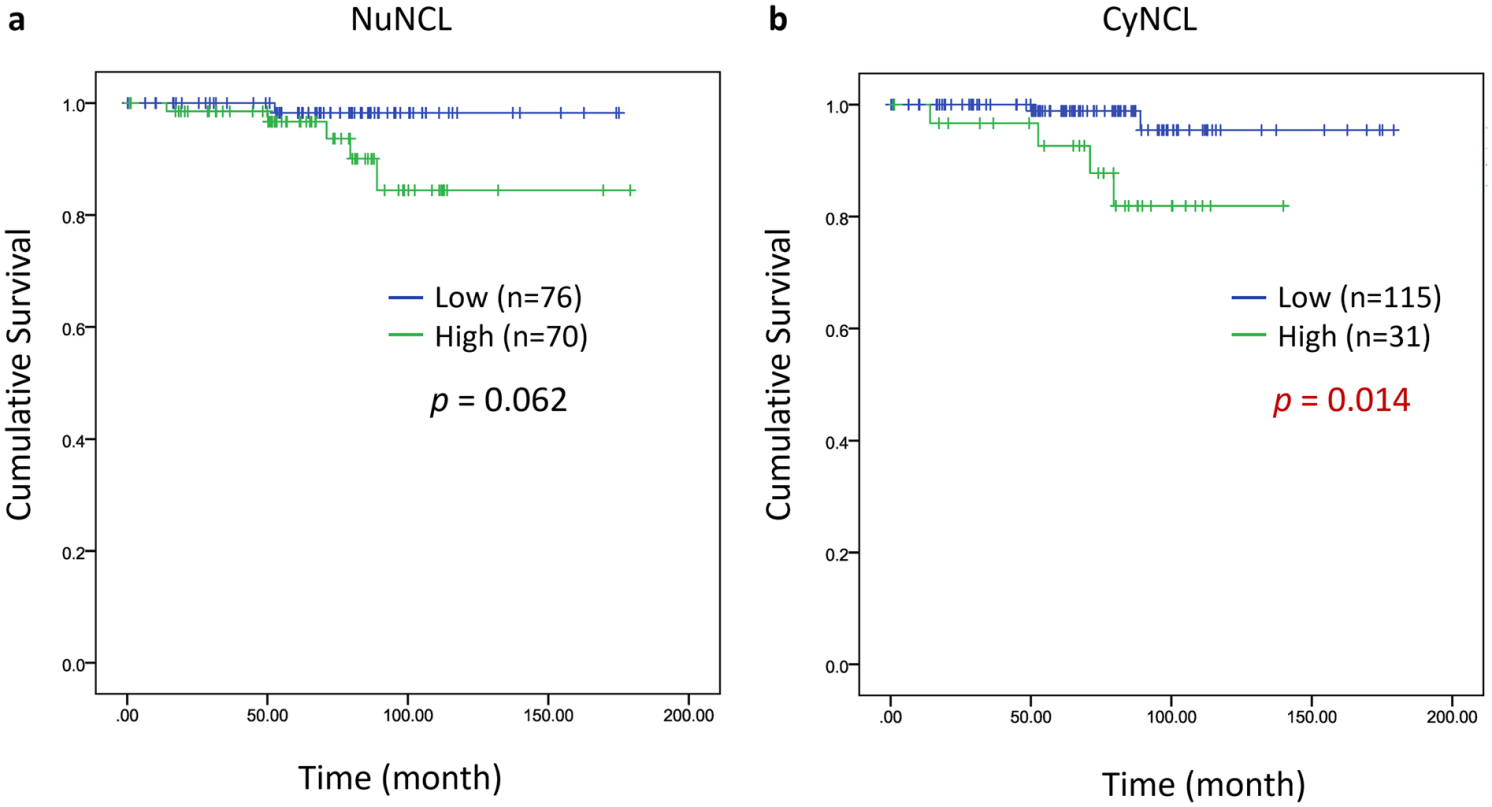
Kaplan–Meier analysis (log rank test) of **a** NuNCL and **b** CyNCL in TNBC cases

We have recently reported the total NCL in clinical samples and its correlation with poor prognosis in TNBC patients [45]. From the same set of patient samples, we analyzed NuNCL and CyNCL and performed the KM analysis. A significant association between CyNCL and poor survival patient was observed (*p* = 0.014), while no significant correlation between NuNCL and patient survival was observed (Fig. 7). These findings support the current meta-analysis where CyNCL, but not NuNCL, was associated with poor prognosis in TNBC patients. Interestingly, no memNCL was detected in these samples.

## Discussion

NCL is overexpressed in a variety of cancers. Overexpression of NCL mRNA was a marker of poor OS and DFS in triple-negative breast cancer [48], acute myeloid leukemia [49], and neuroblastoma [50]. Moreover, overexpression of total NCL protein detected by IHC was reported to be significantly associated with poor OS [15, 16, 18] and DFS [13–15, 47] in pediatric ependymoma, hepatocellular carcinoma, pancreatic ductal adenocarcinoma, and non-small cell lung cancer, but no significance with patient outcome was reported in the studies with regards to ependymoma, hepatocellular carcinoma, and pancreatic ductal adenocarcinoma [14, 19, 20]. Three studies reported an association with cytoplasmic NCL expression and poor prognosis in endometrial carcinoma, gastric cancer, and non-small cell lung cancer [21, 22, 47], compared to nuclear NCL being associated with good prognosis [21–23]. In the current study, a meta-analysis was performed to investigate the impact of different subcellular NCL localization as prognosis markers for cancer patients to provide supporting evidence for differing NCL cellular localization being associated with different patient outcome. The results using HR values as the predictive markers reveal that cytoplasmic NCL is a strong predictive marker for short patient OS, and both cytoplasmic NCL and total NCL are associated with short patient DFS. Interestingly, the nuclear NCL represents the predictive marker for patient long OS and DFS.

The different functions of subcellular NCL have been reported [27], and the potential mechanism of each subcellular NCL is summarized in Fig. 1. The depletion of NuNCL resulted in a decrease pre-rRNA and was associated with rDNA heterochromatinization [51]. NCL overexpression, on the other hand, caused an increase in pre-rRNA levels [51]. NCL has been shown to interact with rDNA chromatin [29], particularly the promoter and coding region of unmethylated rRNA genes [51]. The binding of NuNCL to rDNA inhibited the binding of thyroid transcription factor 1 (TTF-1) to the promoter-proximal terminator T0. Because TTF-1 binding is required for histone deacetylase (HDAC) recruitment, it was proposed that NCL inhibited the formation of repressive heterochromatin and required for the maintenance of a euchromatin active state, promoting active transcription of rDNA [51]. Furthermore, NuNCL forms a complex with replication protein A (RPA), a ssDNA binding protein that is required for DNA replication initiation and elongation. As a result, RPA sequestration by NCL may prevent DNA replication [52]. The cytoplasmic NCL interacted with some RNAs supporting their stability and translation. The cytoplasmic NCL recognized the AU-rich element (AUUUA) in the 3’ UTR of *Bcl-xl* mRNA [53]. This interaction protected *Bcl-xl* mRNA from nuclease degradation [53]. NCL also bound to the 3’ UTR of *Bcl-2* mRNA, increasing its stability and allowing tumor cells to escape the apoptotic pathway [54]. CyNCL bound to the 5’ UTR of *p53* mRNA and inhibited its translation allowing tumor cells to avoid apoptosis [27]. Membrane NCL interacted with Fas and blocked Fas-FasL interaction preventing Fas-mediated apoptosis [40]. In addition, NCL interaction with ErbB1 [55] and Ras [56] at the plasma membrane favored cell proliferation. Moreover, membrane NCL interaction with Ras-GTP increased the interaction of NCL with ErbB1 leading to an accumulation of Ras-GTP. NCL, ErbB1, and Ras acted synergistically in mediating tumor growth in nude mice [56] and in favoring cancer cell proliferation and survival in vitro in human colon cancer cells and prostate cancer cells [41].

Hence, the papers included in the current study were divided into three groups based on the results of the full-text screening: total, cytoplasmic, and nuclear NCL expression. Interestingly, membrane NCL was reported to be associated with cancer progression and explored as a target for cancer treatment in the clinical trials using various molecules such as AS1411 [57]. The membrane NCL data cannot be separately analyzed for its prognostic value as the result of no membrane NCL reported in the recruited articles in this meta-analysis study.

When compared to total NCL expression, high cytoplasmic NCL expression had a strong prognostic value for OS, whereas total and cytoplasmic NCL expressions had the same prognostic value for DFS. These findings can be supported by the previous report of the cytoplasmic NCL function to promote cell proliferation, anti-apoptosis, tumor migration, and metastasis leading to the aggressive phenotypes of cancer cells [9]. Hence, it is strongly suggested that cytoplasmic NCL is a high-impact marker for short survival time in cancer patients. Using cytoplasmic NCL in combination with total NCL or cytoplasmic + nuclear + total NCL can predict bad prognosis. Interestingly, only cytoplasmic NCL is the most potent marker for aggressive cancer which may require aggressive treatment and intensive follow-up. Our result from the TNBC cases confirms the prognostic value of CyNCL as was associated with poor prognosis in TNBC patients.

High expression of NuNCL exhibited the statistically significant marker for long OS, even though no significant correlation with patient long DFS. This could be explained by NuNCL regulating gene transcription, DNA replication, and DNA repair [33–36], resulting in low capability of cancer cell to grow and survive. Though the proposed functions of NCL in the nucleus to control angiogenesis which can then induce aggressive cancer resulting to shot survival time, several mechanisms involved in new vessels formation [30]. Therefore, NuNCL may have major function in controlling gene expression involved in inhibiting cell proliferation. A future study involving target genes controlled by NuNCL is required to explore and better understand how NuNCL contributes to good prognosis in cancer patients.

Total NCL has been reported, which does not specify which cellular compartment NCL is predominantly expressed in. Therefore, the representative images of NCL protein expression were carefully examined and on examining the images of the published papers recruited in this study, only half of the papers displayed NuNCL; the remaining were observed to have both NuNCL and CyNCL expression, with the exception of one paper where only CyNCL was observed. As NuNCL and CyNCL, supported by our findings and functional studies, demonstrate distinct function and prognosis value, the subcellular NCL score should be considered separately for use as a cancer prognosis marker rather than using total scoring.

The overall results emphasize the prognostic value of subcellular NCL as a potential cancer prognosis marker. However, there are two main limitations to this study: (1) There was a high heterogeneity among the studies, which could be attributed to the pooling of data sets from various cancer types due to the limited number of NCL studies in each cancer and (2) the lower precision of HR due to the extraction method rather than directly acquired from the original data. To validate these findings, a larger and well-characterized patient cohort from the same cancer type, employing the same antibody, is required.

## Conclusion

The finding from this meta-analysis supports the observation that high NCL expression is associated with poor prognosis in cancer patients including ependymoma, hepatocellular carcinoma, non-small cell lung cancer, pancreatic ductal carcinoma, endometrial carcinoma, gastric cancer, and B cell lymphoma. We propose herein, using subcellular localization of NCL is more suitable as a prognosis marker in cancers than the total NCL. High CyNCL expression is a prognosis marker for short survival time, whereas high NuNCL expression is a potential prediction marker for prolong survival in cancer patient. However, further cohort studies are required to support this conclusion; this information highlights the importance of different cellular localization of NCL with different proposed functions leading to the potential of being predictive marker for bad or good prognosis in cancer patients.

## Data Availability

The datasets used and/or analyzed during the current study are available from the corresponding author on reasonable request.
